# Wearable Soft Microtube Sensors for Quantitative Home-Based Erectile Dysfunction Monitoring

**DOI:** 10.3390/s22239344

**Published:** 2022-11-30

**Authors:** Chee Ming Noel Sng, Li Min Camillus Wee, Kum Cheong Tang, King Chien Joe Lee, Qing Hui Wu, Joo Chuan Yeo, Ali Asgar S. Bhagat

**Affiliations:** 1 Institute for Health Innovation and Technology (iHealthtech), National University of Singapore, Singapore 119276, Singapore; 2 Department of Urology, National University Hospital, Singapore 119074, Singapore; 3 Department of Biomedical Engineering, National University of Singapore, Singapore 117583, Singapore

**Keywords:** erectile dysfunction, nocturnal penile tumescence, axial rigidity, tumescence, buckling force, Young’s modulus, microtubular sensor

## Abstract

Quantifiable erectile dysfunction (ED) diagnosis involves the monitoring of rigidity and tumescence of the penile shaft during nocturnal penile tumescence (NPT). In this work, we introduce Erectile Dysfunction SENsor (EDSEN), a home-based wearable device for quantitative penile health monitoring based on stretchable microtubular sensing technology. Two types of sensors, the T- and R-sensors, are developed to effectively measure penile tumescence and rigidity, respectively. Conical models mimicking penile shaft were fabricated with polydimethylsiloxane (PDMS) material, using different base to curing agent ratios to replicate the different hardness properties of a penile shaft. A theoretical buckling force chart for the different penile models is generated to determine sufficiency criteria for sexual intercourse. An average erect penile length and circumference requires at least a Young’s modulus of 179 kPa for optimal buckling force required for satisfactory sexual intercourse. The conical penile models were evaluated using EDSEN. Our results verified that the circumference of a penile shaft can be accurately measured by T-sensor and rigidity using the R-sensor. EDSEN provides a private and quantitative method to detect ED within the comfortable confines of the user’s home.

## 1. Introduction

Penile health is an important part of men’s health which significantly impacts their quality of life. Penile disorders such as Erectile Dysfunction (ED) are one of the most under-reported medical conditions. It is estimated that 70% of patients do not seek medical attention due to privacy reasons and social stigma [1]. Furthermore, ED affects between 10 and 25% of men worldwide with the onset risk correlated with increasing age, chronic medical conditions, psychological effects, and lifestyle choices [2]. It was estimated that between 1995 and 2025, the number of men likely to be affected by ED globally would have more than doubled to 322 million men [3].

ED is defined as the consistent inability to attain or maintain a penile erection sufficient for satisfactory sexual intercourse [4]. Quantifiable ED diagnosis includes monitoring of both rigidity and tumescence which is the hardness and expansion of the penile shaft, respectively, during nocturnal penile tumescence (NPT). NPT refers to erections that commonly occur during the rapid eye movement (REM) sleep stage. The term rigidity includes the measurement of either axial or radial rigidity—Timm et al. [5] have verified that measurements of radial rigidity are physically equivalent to axial rigidity. For evaluation, it is best to include the measurement of axial rigidity expressed in grams of force required to buckle a penile shaft (buckling force) as it correlates most accurately with the ability to achieve vaginal penetration [6].

ED diagnosis has been studied extensively with the development of commercially available medical devices such as Snap*Gauge^®^ band [7] and Rigiscan^®^ [8]. The Snap*Gauge^®^ band is widely used as a screening device due to its low-cost and ability to provide rigidity estimates. However, it does not provide a quantitative analysis that includes the duration and number of erections per night [9]. While Rigiscan^®^ provides quantified ED diagnosis, it suffers from a high probability of registering either false negatives or false positives, with highest diagnostic accuracy around 75.9% [10,11]. To have a more detailed diagnosis, patients are required to be in a sleep laboratory that offers concurrent measurement of penile activity including REM sleep for consecutive nights with tethered bulky diagnostic tools which in turn impacts patient comfort [12,13].

There have been several attempts to develop a real-time ED diagnosis device that can be privately operated in the comfort of the patient home [14]. Similarly, for the diagnostic evaluation of ED, there are attempts to investigate using different diagnostic methods such as Doppler ultrasonography and shear wave elastography to better evaluate ED or penile rigidity instead of using the traditional RigiScan^®^ device [15,16]. Notwithstanding the recent advances in diagnostic methods, limitations remain for ED or vascular ED diagnosis [10,17], with testing in a clinical setting almost indispensable.

We believe more can be done to address issues on privacy and social stigma so that ED patients will be reassured to seek early medical attention as studies have found that this malady can be reversible in many instances provided it is treated at an early stage [18]. With this in mind, we developed a wearable device equipped with flexible sensors for quantitative yet accurate penile health monitoring.

**Figure 1:**
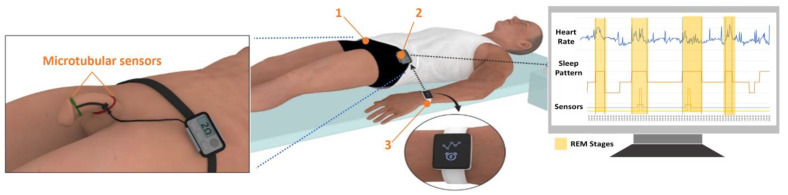
Illustration of the concept of EDSEN consisting of (1) a pair of microtubular sensors, (2) a wireless wearable detector, and (3) a smartwatch for REM sleep monitoring.

In this work, we introduce Erectile Dysfunction SENsor (EDSEN), a home-based wearable device for quantitative penile health monitoring which utilizes stretchable tubular sensors worn around the penile shaft [19]. EDSEN consists of a pair of microtube sensors, a wireless wearable detector, and a smartwatch for sleep monitoring (Figure 1). To operate EDSEN, the patient will wire the sensors to a detector worn around the adjacent waist region with the microtubular sensors firmly secured around the penile shaft. A smartwatch will be worn on the wrist for sleep stage monitoring. A healthy person will experience on average three to four erections during a full night’s sleep [20], with each erection lasting about 25 min [21].

In order to investigate the different penile circumferences and rigidity, conical polydimethylsiloxane (PDMS) models mimicking penile shafts were fabricated to have different Young’s moduli by adjusting the amount of curing agent used. This work validates EDSEN’s capability to quantitatively measure ED and will pave the way for a smart wearable home-based device that can accurately monitor penile health, address privacy issues, and ultimately empower more men to seek medical treatment.

## 2. Materials and Methods

### 2.1. EDSEN Device

#### 2.1.1. Microtube Sensors

**Figure 2:**
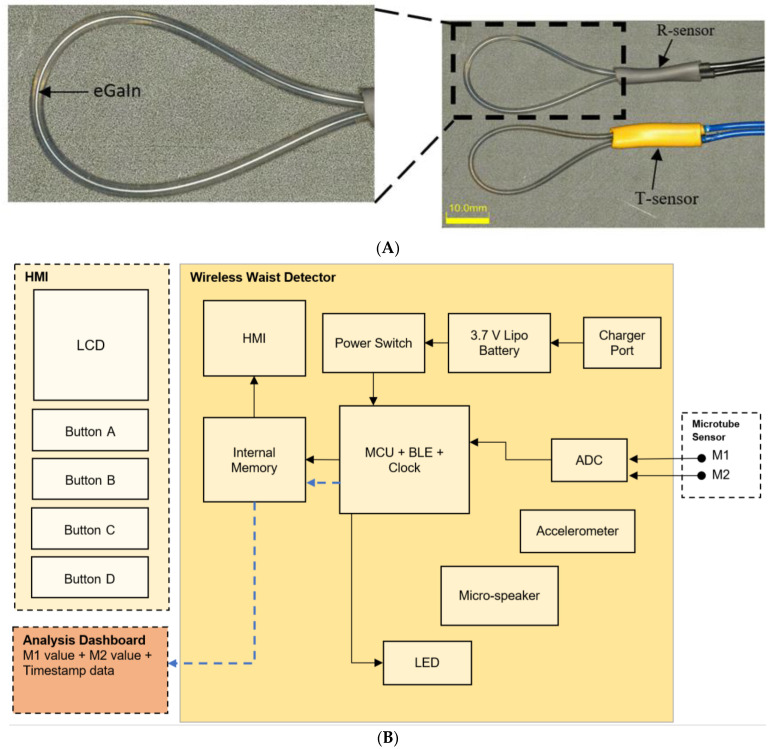
(**A**) Loop-design microtube R-sensor (top) and T-sensor (bottom). (**B**) Electronics system layout and architecture of the waist detector.

The fabrication approach of the microtubular sensors is detailed in Xi et al. [19]. Briefly, hollow microtubes were mounted on a customized vertical extrusion jig and injected with liquid metal, eutectic gallium indium (eGaIn). The microtubular sensors are fabricated with similar material properties as commercial latex which is traditionally used to manufacture condoms. The temporal and spatial resolution of the microtubular sensor are 20 milliseconds and 0.3 centimeters, respectively. For this work, we redesigned the stretchable sensors into a loop design (Figure 2) to fit the typical girth of a penile shaft. These microtubular sensors are soft, flexible, and easy to use, enabling the patient to effortlessly wear or stretch the sensors onto the penile shaft.

To quantitatively measure both tumescence and rigidity using our loop-design microtubular sensors, we fabricated them such that they have different Young’s moduli to effectively measure their corresponding electrical resistance upon stretching. Two types of microtubular sensors were fabricated, the R-sensor with Young’s modulus of 1.63 MPa measures rigidity, and the T-sensor with Young’s modulus of 0.39 MPa measures tumescence of the penile shaft (Figure 2A).

#### 2.1.2. Wireless Wearable Detector

Our wireless waist detector is made up of a Printed Circuit Board (Funambolo Technologies, Bengaluru, India) that was designed and fabricated to provide the following functions: first, measure and record the resistance value of the T- and R-sensors with a resistance range between 0 to 10 Ω ± 0.1 and second, transfer raw temporal data picked up by both sensors from waist detector to analysis dashboard via Bluetooth Low Energy (BLE) connection (Figure 2B). The wireless waist detector is an independent unit which works on its own with an embedded memory chip and lithium-ion battery, it is rechargeable via micro-USB and is able to record and store up to eight hours of data. There are two additional ports to connect the microtubular sensors and read data.

The analysis dashboard is a customized software interface developed for data processing of the EDSEN device. Following the extraction of the raw data, it can be saved and processed in various formats for further analysis.

**Figure 3:**
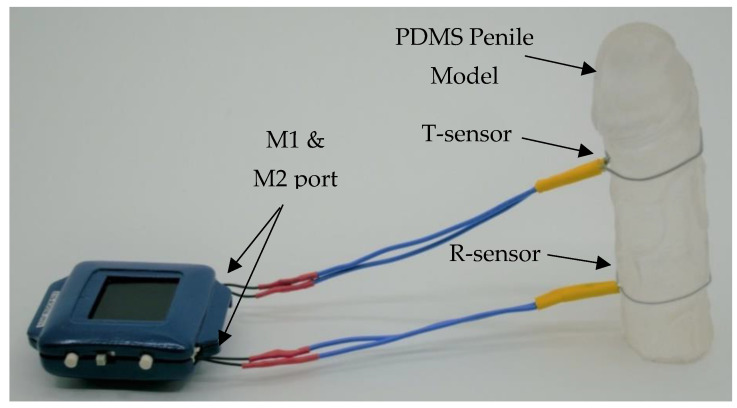
Illustration of the waist detector and microtubular sensors in operation around a dummy PDMS-based penile model.

To operate the device, the wireless waist detector will be connected via BLE to the analysis dashboard for time synchronization and both sensors must be connected via the M1 and M2 ports. Once the sensors are connected, they can strap onto the penile model for evaluation (Figure 3). After testing, raw data can be imported and processed via the analysis dashboard.

### 2.2. Fabrication of Penile Shaft Models

**Figure 4:**
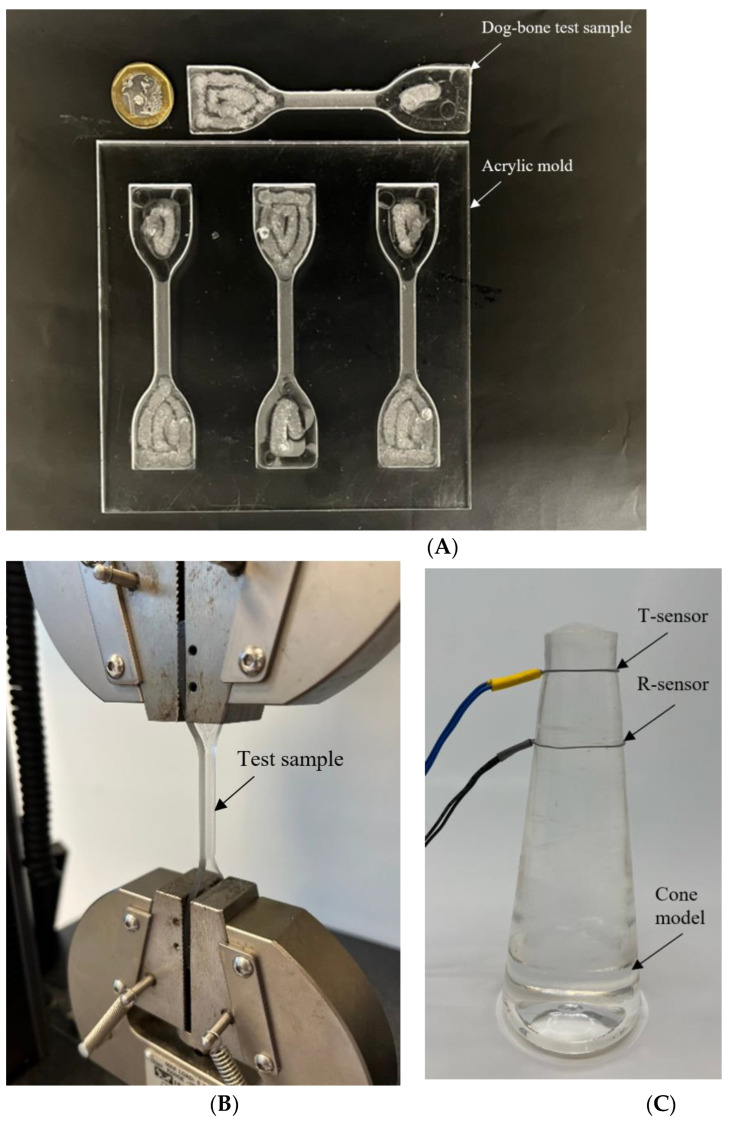
(**A**) Acrylic mold (bottom) and a PDMS dog-bone test sample with dimensions in accordance with ASTM standard (top). (**B**) Illustration of test apparatus clamping a dog-bone tensile sample piece. (**C**) Cone shape penile model.

To mimic the different mechanical properties of a flaccid and erect penis, we fabricated a conical shape PDMS (Dow Corning Corporation, Sylgard 184, Midland, MI, USA) model with a different base to curing agent ratios (5:1, 10:1, 20:1, 30:1, and 40:1). The cone model is used to mimic the properties of a penile shaft with circumference ranging from 8 to 14 cm which covers the 5 to 95 percentiles of penile circumference [22]. Microtube sensors are secured onto the cone shape models (Figure 4) to investigate and present quantitative data regarding the change in resistance with different circumferences and rigidity.

To fabricate a cone model, Sylgard 184 silicone elastomer, supplied as a two-component kit, was prepared by mixing the pre-polymer base (part A) and cross-linking curing agent (part B) at room temperature and degassing the mixture in a vacuum desiccator for 30 min. To avoid re-introducing air bubbles, the degassed PDMS mixture was then gently poured into a cone shape mold pre-coated with mold release agent (Weicon, Formentrennmittel, Münster, Germany), and left inside a 70 °C curing oven (Binder, ED 53) for two hours.

### 2.3. Fabrication of Dog-Bone Test Samples

To find out the different material properties of PDMS prepared with different mixing ratios, the test samples of PDMS were cast from dog-bone shape acrylic molds machined with a CNC miller (Datron NEO, Milford, NH, USA) in accordance with the respective American Society for Testing of Material (ASTM) standards for tensile testing of rubber and elastomeric materials.

ASTM D412 Type C standard was used to manufacture the test samples which specifies a dog-bone shaped test structure, whereby the wider corners are clamped into the test apparatus while the narrow neck region is tested (Figure 4). Sample pieces were cast from a 3.0 mm thick Acrylic (Figure 4) mold.

Similar to the fabrication of the conical model, the degassed PDMS-filled molds were placed in an oven to cure at 70 °C for two hours. After curing, any excess and overflow PDMS mixture was removed with a razor blade before testing.

Tensile testing of samples (Figure 4) was performed on a universal tester machine (Instron Dual Column Model 5965, Instron). The crosshead velocity was set to 500 mm/min, and a minimum of three test samples were individually loaded for each mixing ratio. The test results—Young’s modulus, tensile stress, and tensile strain—were automatically calculated and logged by the tester software and manually saved (BLUEHILL^®^, Instron, Norwood, MA, USA).

## 3. Results and Discussion

### 3.1. Tumescence Measurement

To ensure that the reading of our M1 and M2 ports was accurate, we compared our results against a Digital Multimeter (DMM) reader. At least five different microtube sensors were tested to ensure reproducibility. Both T-sensor and R-sensor are used to verify by connecting them individually to the DMM, the M1 port, and the M2 ports, respectively. The sensors were secured onto a PDMS cone model to measure the resistance for increasing circumference values.

**Figure 5:**
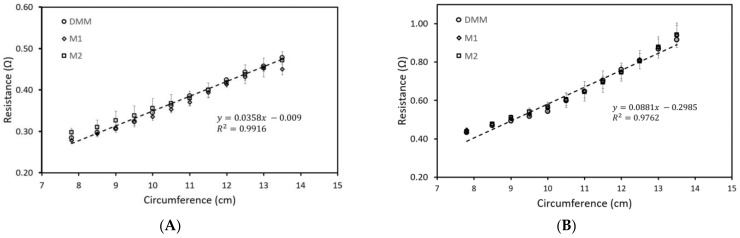
Verification of the resistance of T-sensor (**A**) and R-sensor (**B**) for increasing circumference (penile diameter) measured via ports M1, M2, and DMM.

The resistance value of both the T-sensor and R-sensor for the different circumferences are shown in Figure 5. As expected, the resistance displays a linear relationship to circumference with an R-square of 0.99 and 0.98 measured by our EDSEN waist detector (ports M1 and M2) as well as the DMM for both the T-sensor and R-sensor, respectively. These results indicate that the microtube sensors can measure the tumescence of a penile shaft with high sensitivity.

### 3.2. Rigidity Measurement

Rigidity can be determined from either axial or radial rigidity. Although they are physically equivalent [5], axial rigidity correlates with the ability of an erect penile shaft to resist deformation and achieve vaginal penetration [23]. For this study, we evaluated the axial rigidity of our penile model in terms of buckling force and correlated it with the criteria for sexual intercourse (Table 1).

The engineering analysis of buckling force (*F_buc_*) determined by Udelson et al. [24] is derived from Euler’s formula for ‘critical load’ and is defined as:

(1) $$F_{buc}=\frac{\pi^{2}EI}{L^{2}}$$

where *E* is Young’s modulus of elasticity, *I* is the second moment of area of the column cross-section about its neutral axis, and *L* is Length. *I* is defined as:

(2) $$I=\frac{{\pi D}^{4}}{64}$$

where D is the diameter.

We adapted the buckling force and its corresponding criteria for sexual intercourse determined by Virag et al. [25] and defined it as:

**Table 1:** Summary of buckling force (kg) and its corresponding criteria for sexual intercourse adapted from Virag et al. [25].

Buckling Force (kg)	Criteria (for Sexual Intercourse)
<0.5	Insufficient
0.5–1	Sufficient
>1	Optimal

The assumptions for our theoretical model include an average erect penile length of 13 cm [22] and a penile diameter from 2.3 to 4.5 cm with a corresponding circumference of 7.2 to 14.1 cm in circumference. We determined the Young’s modulus for each penile model individually, calculated the buckling force of each model, and related them to each of its criteria shown in Table 1. The minimal buckling force required for sexual intercourse is 0.5 kg, any buckling force above 0.5 kg will be sufficient for sexual intercourse.

**Figure 6:**
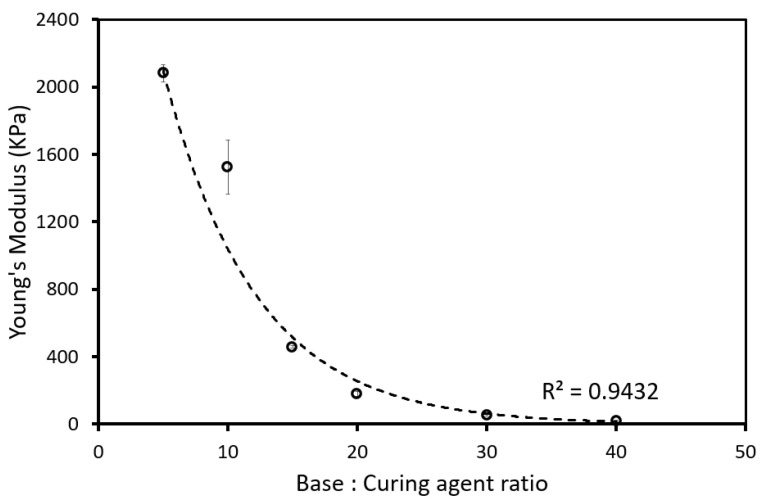
Relationship between the different mixing ratios and the resultant Young’s modulus.

To establish the relationship between the base-to-curing agent ratio and Young’s modulus of our penile models, we tested different mixing ratios using the dog-bone-shaped test samples previously fabricated shown in Figure 4. Each data point in Figure 6 represents the average Young’s modulus of three dog-bone shaped test samples for each mixing ratio with a 5:1 ratio having the highest modulus. The relationship between Young’s modulus and base to curing agent ratio is non-linear and consistent with other studies [26,27].

The results in Figure 6 reflect the different mechanical properties of the test samples. A mixing ratio of 5:1 will lead to a rigid and stiff penile shaft model and for 40:1, a soft and flaccid model. Further analyses were carried out based on these Young moduli to calculate the buckling force of penile shafts with varying rigidity and relate them to the criteria for sexual intercourse (Table 1).

### 3.3. ED Testing

**Figure 7:**
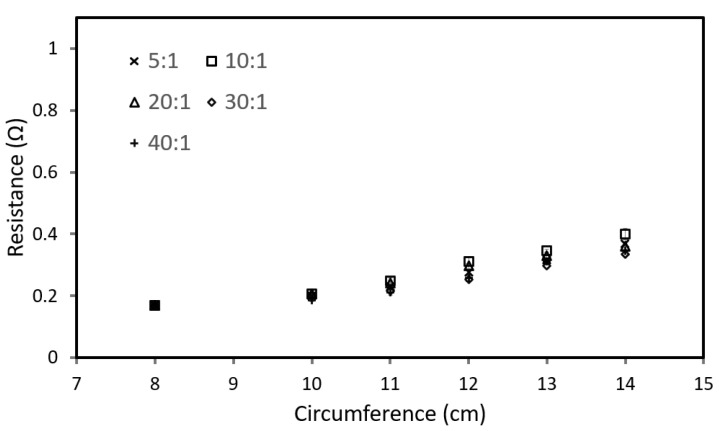
Comparison of resistance value for T-sensors with different mixing ratios.

**Figure 8:**
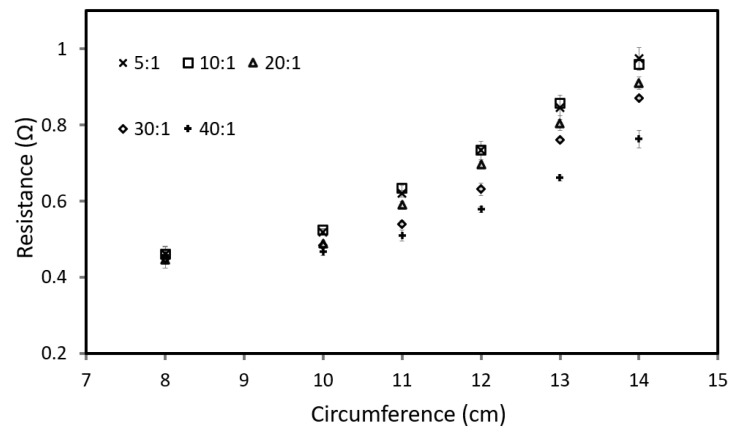
Comparison of resistance value for R-sensors with different mixing ratios.

For this test, both T- and R-sensors are looped around various PDMS cone models. Figure 7 and Figure 8 show the resistance value of the T-sensor and R-sensor, respectively, determined by averaging the results from six different microtube sensors.

Figure 7 illustrates a comparison of the T-sensor resistance values across different PDMS cone models. We observed the resistance values of the T-sensor are almost indistinguishable across all mixing ratios (models with different rigidity). As circumference increases, the T-sensor reading increases linearly irrespective of rigidity. This is because T-sensors are comparatively softer microtubes with Young’s modulus of 0.39 MPa, it does not apply much compression once expanded and will measure the corresponding circumference of the model (correlating tumescence).

Figure 8 shows a comparison of the R-sensors resistance value for the different PDMS penile shaft models. The results in Figure 8 demonstrate that the R-sensors are able to identify models of varying rigidity, with the distinction becoming sharper as the circumference increases. This is because R-sensors are made up of harder material with Young’s modulus of 1.63 MPa, and applies a much higher compression force when expanded and will measure the ‘stiffness’/rigidity of the model.

When both sensors are used together (Figure 3), we can measure the circumference from the T-sensor and determine the rigidity of the penile shaft from the R-sensor readings. However, the results for mixing ratios 5:1 and 10:1 are relatively similar, and this can be due to the elastic modulus of PDMS with a base-to-curing agent ratio of 10:1 and lower, which is dependent on the crosshead speed [28].

### 3.4. Buckling Force Analysis

**Figure 9:**
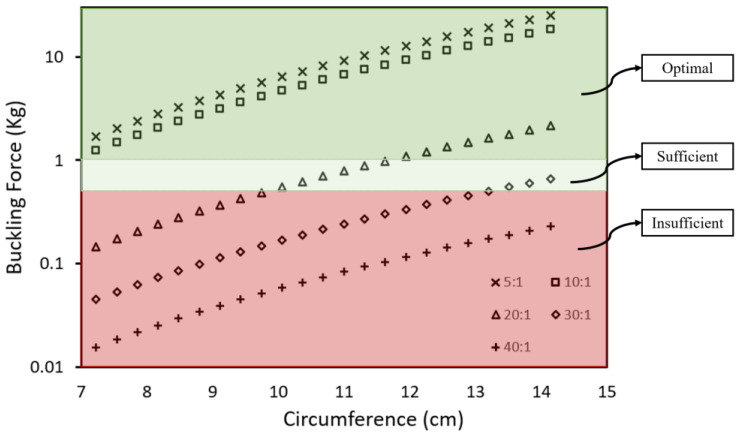
Relationship between the circumference and the resultant buckling force of the different penile models.

We derived the buckling force for each mixing ratio by maintaining the length value of Equation (1) to that of an average erected penile length [22] while substituting the different values of Young’s modulus and penile diameter. Figure 9 shows the theoretical buckling forces for different PDMS models for which we have color-coded into different zones to match each sufficiency criterion. To accurately measure the buckling force of a penile shaft, all three parameters, circumference, Young’s modulus, and length, are required and a single parameter alone does not determine the sufficiency criteria. Our results show that with an average penile length, the PDMS model with a mixing ratio of 40:1 has an insufficient buckling force (<0.5 kg, red zone) regardless of its circumference (from 7.2 cm circumference onwards), which indicates a flaccid penile state. Both mixing ratios of 5:1 and 10:1 have an optimal buckling force (>1 kg, green zone) regardless of the circumference (from 7.2 cm circumference onwards) which indicates a rigid penile state. We infer that a penile shaft with Young’s modulus similar or below mixing ratio 40:1 indicates a flaccid state and will always be within the insufficient criterion even with a circumference of 14 cm, this is consistent with Cheng et al.’s study on Young’s modulus at different degree of erection hardness [29].

Results of the 30:1 model show that the penile shaft remains in the insufficient sexual intercourse region until a circumference of 13 cm. Penile shafts of similar rigidity, but a larger diameter (circumference) are capable of sexual intercourse as the buckling force is sufficient (>0.5 kg, <1 kg, light green zone). This means that the penile shaft will be insufficient for sexual intercourse before a circumference of 13.5 cm. The 20:1 model has sufficient buckling force from 10 cm circumference and enters the optimal range from 12 cm circumference.

For an average erect penile length and circumference, 13 cm and 12 cm, respectively [22], we require at least 179 kPa Young’s modulus to have an optimal buckling force for sexual intercourse. Our theoretical model uses an average erected penile length in all calculations plotted in Figure 9; however, it is good to note that variations in the penile elongation can result in a significantly different buckling force value as length is inversely proportional to buckling force.

## 4. Conclusions

We performed a series of verification tests on microtube sensors to investigate their working relationship with penile circumference. Further, we performed tensile tests to examine the material properties of Sylgard 184 which differ over a range of base to curing agent ratios. From these tests, we substantiated the results of our microtube sensors, determined the resultant range of Young’s modulus, and ascertain the different buckling forces of each mixing ratio.

We observed a linear relationship between the microtube sensors resistance and penile circumference, and an exponential relationship between Young’s modulus and mixing ratio. This is evidenced by a significant drop in Young’s modulus from 2.079 to 0.019 MPa that corresponds to an increase in mixing ratio from 5:1 to 40:1. Taking advantage of this decrease in mechanical stiffness, we will be able to replicate penile models of different rigidities with Sylgard 184 to determine the theoretical buckling force of each model.

With our T- and R-sensors, we evaluated different penile models and demonstrated the suitability of the T-sensor to determine the circumference of the penile shaft. When used together with the R-sensor, we are able to then deduce the rigidity by matching the R-sensor resistance and circumference values. If successfully matched, the theoretical buckling force can be calculated, and with the erect penile length known, ED can be evaluated.

With EDSEN, we demonstrated the ability to quantitatively measure penile tumescence and rigidity. We created a theoretical buckling force graph to predict the criteria for sexual intercourse for each penile model based on different penile diameters, lengths, and Young’s modulus. This study serves as a basis for future design improvements of EDSEN and possibly other smart wearable devices that leverage microtubular sensors for penile health monitoring. Future development of EDSEN will include usability, acceptability evaluation, and additional measurement of physiological parameters such as length.

## Data Availability

Not applicable.
